# Crystal Structure Analysis of the First Discovered Stability-Enhanced Solid State of Tenofovir Disoproxil Free Base Using Single Crystal X-ray Diffraction

**DOI:** 10.3390/molecules22071182

**Published:** 2017-07-14

**Authors:** Ji-Hun An, Alice Nguvoko Kiyonga, Woojin Yoon, Hyung Chul Ryu, Jae-Sun Kim, Chaeri Kang, Minho Park, Hoseop Yun, Kiwon Jung

**Affiliations:** 1 College of Pharmacy, CHA University, Sungnam 13844, Korea; ajh@chauniv.ac.kr (J.-H.A.); gabriella@chauniv.ac.kr (A.N.K.); kchaeri9292@naver.com (C.K.); minho.park92@gmail.com (M.P.); 2 Department of Chemistry and Energy Systems Research, Ajou University, Suwon 16499, Korea; xtal@ajou.ac.kr; 3 R&D center, J2H Biotech, Ansan 15426, Korea; daman-ryu@j2hbio.com (H.C.R.); jsbach@j2hbio.com (J.-S.K.)

**Keywords:** Tenofovir disoproxil, crystal structure, stability, solubility, active pharmaceutical ingredients

## Abstract

Tenofovir disoproxil (TD), an anti-virus drug, is currently marketed under its most stable form, Form-I of Tenofovir disoproxil fumarate (TDF). However, studies regarding the properties of TD free base crystal as a promising drug as well as its crystal structure have not yet been reported. This assumption was made because TD free base is not directly produced in a solid form during the manufacturing process. TD free base is first obtained in an oil form, and is then synthesized into TDF crystal. In this regard, the present study was conducted to investigate both the potentiality of TD free base to be an active pharmaceutical ingredient (API) and its crystal structure. Here, TD free base solid was produced by means of drowning-out crystallization. Next, single crystal X-ray diffraction (SXD) was employed to determine the crystal structure. Powder X-ray diffraction (PXRD) and a differential scanning calorimetry (DSC) analysis were performed to evaluate the crystal’s properties. Furthermore, experiments were carried out at 15%, 35%, 55%, 75%, and 95% relative humidity (RH) for 12 h using a hygroscopic tester to determine and to compare the hygroscopicity and stability of TD free base with TDF crystal. Additionally, experiments were conducted under accelerated (40 °C, RH 75%) and stress storage (60 °C, RH 75%) conditions for 30 days to investigate the changes in purity and the formation of dimer. In this work, we report that TD free base possesses lower hygroscopicity, and thus does not generate dimer impurity from hydrolysis. Primarily, this is attributed to the fact that TD free base is not an easily ionized salt but comprises neutral hydrophobic molecules. According to the structural properties, the improved hygroscopic property of the TD free base crystal was due to the decrease of crystal polarity owing to the intermolecular H-bonds present in TD free base rings. In addition, the solubility investigation study carried out in aqueous solution and at gastrointestinal pH revealed a similarity in TDF and TD free base solubility under the mentioned conditions. Accordingly, we could confirm the potentiality of TD free base as an active pharmaceutical ingredient.

## 1. Introduction

**Figure 1:**
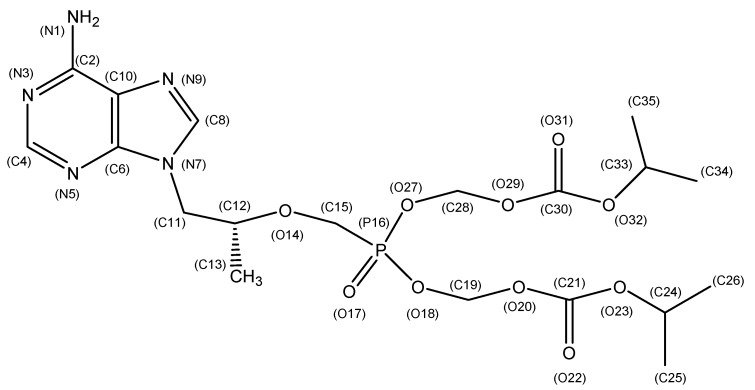
Molecular diagram of the Tenofovir disoproxil (TD) free base with the atomic numbering scheme.

Active pharmaceutical ingredients (APIs) are generally developed in crystalline form, which vary according to the crystal structure. This includes polymorphs, pseudo-polymorphs, salt form, and co-crystals. In addition, it is widely known that the change of crystal structure of a certain API hugely influences its physicochemical characteristics, such as solubility, structural stability, dissolution rate, density, and melting temperature. Such change influences the API’s bioavailability in positive or negative ways. Therefore, studies involving the crystal structure design of APIs are considered imperative for drug development and manufacturing. Accordingly, various strategic studies regarding the design of API crystal structures have been conducted to design a desired crystal structure with improved drug properties [1,2,3]. The crystal structure’s design is achieved using crystallization, a method through which new molecular packing arrangements are induced. The newly designed API crystal structure is analyzed by means of single crystal X-ray diffraction (SXD) [4]. Therefore, several studies involving the use of SXD for the investigation and determination of APIs’ crystal structures have been reported. Park et al. [5] investigated and characterized the crystal structures of four distinct polymorphs of donepezil APIs using SXD. Further, through SXD analysis, Prohens et al. [6] characterized the crystal structure of the new solvate form of adefovir dipivoxil. A study conducted by Actins et al. [7] investigated the crystal structures of the pseudo-polymorphs of the hemihydrate and anhydrate of Droperidol using SXD analysis. In their study, Chandrappa et al. [8], by means of SXD analysis, determined the conformation and intermolecular interaction of the form-I and form-II polymorph structures of a clinical candidate compound, namely Xaliproden (SR57746A). Moreover, being unable to obtain single crystals of form-III, researchers have endeavored to establish the crystal structure using solid-state C-NMR analysis. Accordingly, SXD has been found to be an adequate and the most accurate method for the determination of the characteristics of a particular crystal’s design. A drug used in the present study, Tenofovir disoproxil (TD), which is sold under its product name, Viread^®^, is a bioactive substance with remarkable antiviral activity against the Human Immunodeficiency Virus (HIV) and the Human Hepatitis B Virus (HBV) (Figure 1). The product is marketed as Tenofovir disoproxil fumarate (TDF) salt form, and its scientific appellation is 9-[(*R*)-2[[bis[[(isopropoxycarbonyl)oxy]methoxy]phos-phinyl]-methoxy]propyl]adenine fumarate [9,10]. TDF’s crystal forms include the salts TDF Form-I and ULT-11, and the co-crystals Form-A and Form-B (methanol solvate) [9,10,11,12,13]. The structures of Form-A, Form-B, and ULT-11 have been fully investigated with the use of single crystal diffraction data (Table 1). Yet, the structure of Form-I has not been fully identified until recently. Gomes et al. [10] have reported the structure of Form-I based upon powder diffraction data using the Rietveld technique. However, the reported cell parameters are close to those of Form-A reported by Lee et al. [11], and details of the structure are not fully furnished.

Tenofovir disoproxil (TD) was developed under a TDF form as a prodrug of adefovir dipivoxil (ADV) in 1997 by Gilead Sciences Inc. Form-I is the crystal structure of the developed TDF. In addition, ADV and TD belong to the bis-POC PMPA class. ADV and TD possess a similar molecular structure with few functional group differences. After oral intake, ADV and TD are hydrolyzed at the phosphate vicinity followed by the elimination of the ester group. Here, the drug activity is produced by the ester group free tenofovir (PMPA) for TD and adefovir (PMPA) for ADV [9,14,15,16]. However, ADV and TD hydrolyze in contact with moisture and generate formaldehyde. Formed formaldehyde along with TDF’s amine group induces a condensation reaction, which provokes the formation of an impurity, namely, TD dimer, and consequently decreases the purity of the products [14,15,16]. TDF is a hydrophilic salt, which easily degrades in the presence of moisture. Accordingly, several studies were conducted by Gilead Sciences Inc. (Foster City, CA, USA) to improve the aqueous physicochemical stability and to overcome the drawback of TDF. However, these studies resulted in no improvement [16]. Besides, during the discovery stage of TD in 1997 by Gilead Sciences Inc., TD free base was primarily produced not in a solid form but in an oil form that was then synthesized into TDF [9,17]. Hence, we presume that Gilead Sciences Inc. was unable to yield TD free base solid during the manufacturing stage. Thus, appropriate reference material regarding the crystal structure or physicochemical properties, such as stability and solubility, of TD free base have not been yet reported. Although a TD free base crystal with a melting point at 65 °C was reported by Cipla Limited in 2008, a proper crystal structure analysis or characteristics such as solubility and stability for the drug’s application were not successfully reported [17]. Accordingly, in this study, drowning-out crystallization was conducted on TD free base in oil form to yield a TD free base crystal. The obtained TD free base was found to possess a different crystal structure compared with the TD free base crystal developed by Cipla Limited (Mumbai, India).

Finally, on one hand, this study was performed to analyze and to determine the crystal structure of the TD free base crystal using SXD. On the other hand, this study aimed to overcome the storage stability drawback of TDF as well as to investigate the potentiality of TD free base as an API through a comparative solubility evaluation of TDF with TD free base under a gastrointestinal pH condition.

## 2. Results and Discussion

### 2.1. Crystal Structure Analysis of TD Free Base Crystal Using Single Crystal X-ray Crystallography

The metrical details of the TD free base crystal obtained by means of single crystal X-ray analysis are given in Table 2.

**Figure 2:**
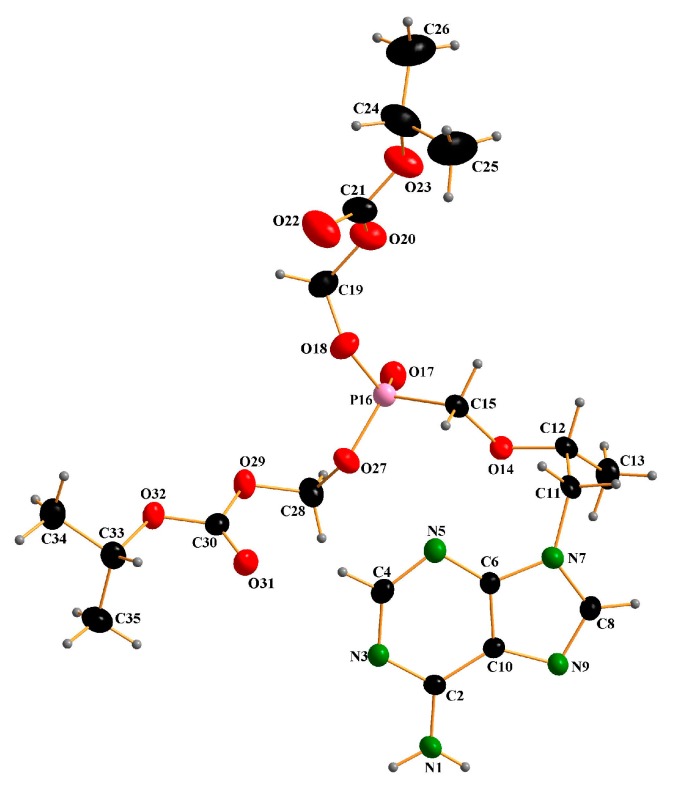
Molecular structure of Tenofovir disoproxil (TD). The displacement ellipsoids are drawn at the 20% probability level.

**Figure 3:**
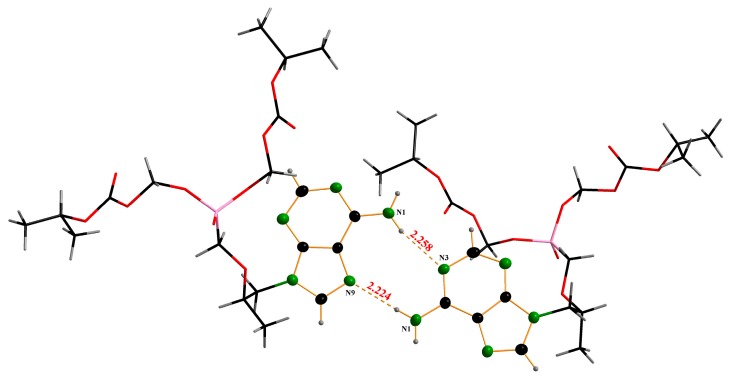
Intermolecular hydrogen bond between nitrogen atoms for Tenofovir disoproxil (TD) free base.

**Figure 4:**
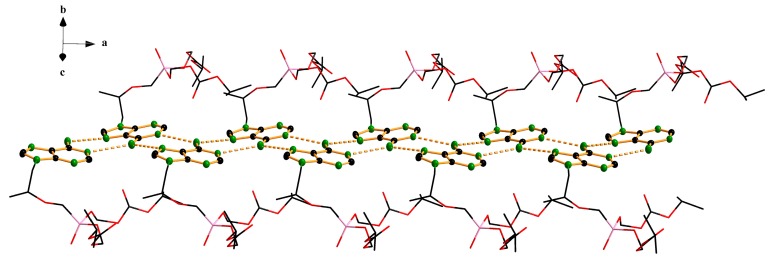
View of a linear chain along the *a*-axis connected via intermolecular hydrogen bonds between the nitrogen atoms of the purine rings.

Figure 1 is a sketch of the TD molecule displaying the numbering scheme. Figure 2 is a drawing of the molecule. The bond distances and angles found in this structure are usual, and they are comparable with those reported from the structural studies on TDF. However, a different conformational structure caused by the free rotation along C11-C12-O14-C15 has been found. The torsion angle of the title compound is 74.5(1)°, while that of Form-A’s structure is 161.5(2)° [11]. Deviations from the planarity of the purine skeleton are rather small, and are comparable with those found in the related structures [11]. It is interesting that the TD molecules are connected through intermolecular hydrogen bonds between the N atoms of the purine rings (N(1)-H---N(3) and N(1)-H---N(9)) (Figure 3, Table 3) to form linear chains along the a-axis (Figure 4). The orbitals of the N atoms involved in the H-bonds are in sp^2^ hybridization, which allows the structure to retain its planarity. As a result, the molecules are connected via in-plane adenine (purine) rings infinitely. There are no unusual inter-chain interactions among the chains except van der Waals forces. To the best of our knowledge, the single crystal structure of TDF Form-I has not been reported yet [12], thus a direct comparison of the two phases is inappropriate. While TDF Form-I contains an acid which can make ionic pairs increase the solubility in water, TD free base is a neutral molecule. We believe that these are responsible for the less hygroscopic nature of the TD free base compared with TDF Form-I. In addition, the intermolecular H-bonds between the purine rings reduce the polarity of TD free base, and this could participate in stabilizing the crystals.

### 2.2. Characterization of TD Free Base Crystal

**Figure 5:**
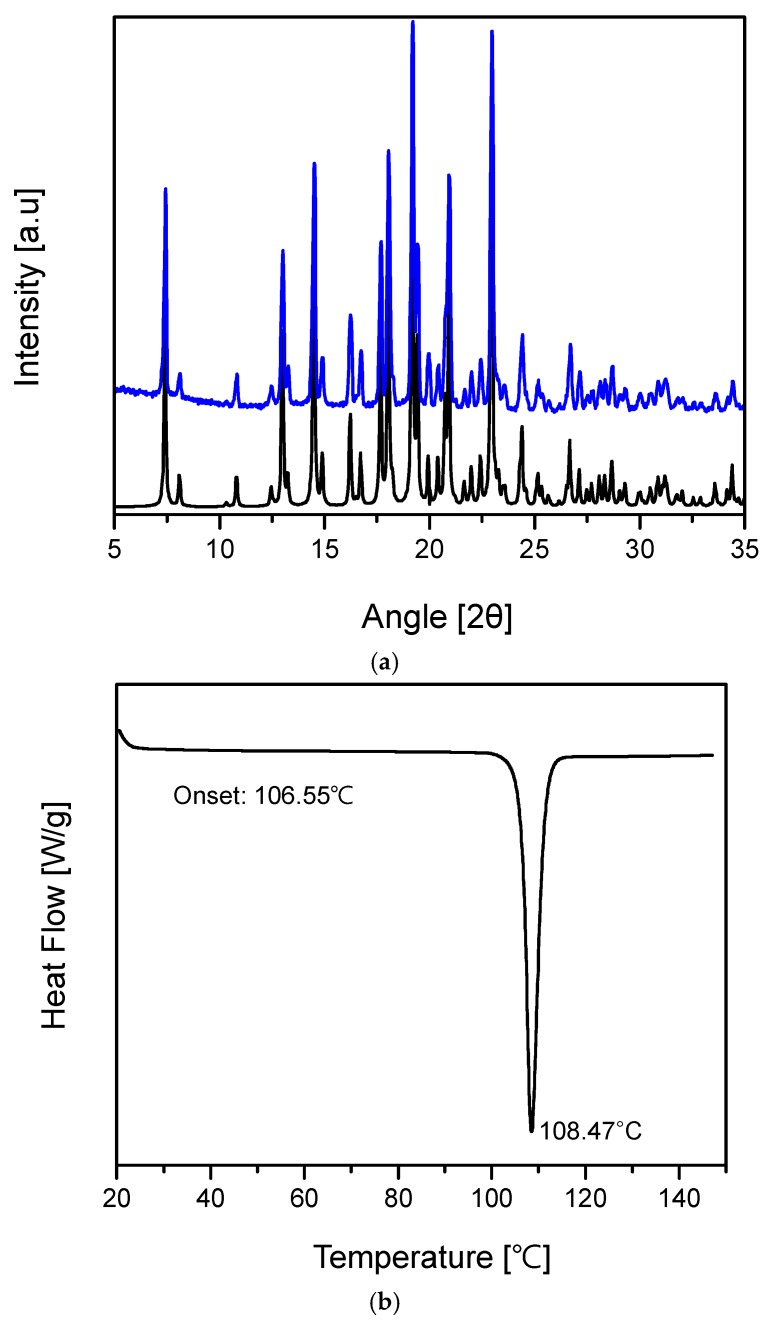
Characterization of TD free base crystal: (**a**) Observed (blue) and simulated (black) powder X-ray diffraction pattern; (**b**) Differential Scanning Calorimetry (DSC) (10 °C/min).

The PXRD results of the free base TD sample clearly show that its structure is different from the reported TDF phases. The PXRD pattern is compatible with the simulated powder diffraction pattern based on the structure determined with single crystal diffraction data, and this is evidence of the purity and crystallinity of the powder sample (Figure 5a). On the other hand, the DSC curve of the new TD free base crystal revealed the endothermic peak’s onset temperature at 106.55 °C, while the endothermic peak was observed at 108.47 °C (Figure 5b).

### 2.3. Hygroscopicity Evaluation of TD Free Base Crystal and TDF Form-I

**Figure 6:**
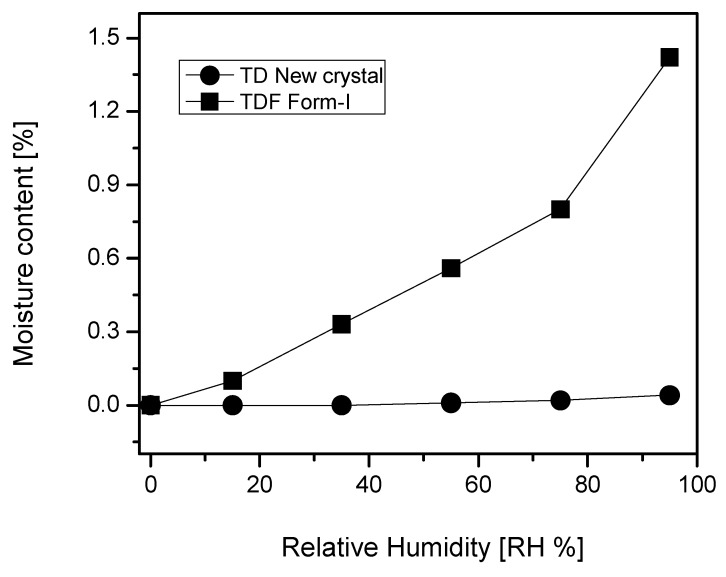
Hygroscopicity evaluation of the new TD free base crystal and TDF Form-I in accordance with the relative humidity (RH) change.

A hygroscopicity analysis was carried out to investigate whether TD free base is adequate to transcend the impurity increment problem due to the hygroscopic property of TDF. Here, the comparative hygroscopic investigation for TDF Form-I and the new TD free base crystal was conducted for 12 h using the hygroscopic tester. As a result, the initial moisture content of 0% was found out to be 0.10% at a relative humidity (RH) of 15%, 0.33% at an RH of 35%, 0.56% at an RH of 55%, 0.8% at an RH of 75%, and 1.42% at an RH of 95%. Namely, a hygroscopicity increase of over 1% could be observed for TDF Form-I. Nevertheless, the moisture content was maintained at 0% at an RH of 15% and 35%, 0.01% at an RH of 55%, 0.02% at an RH of 75%, and 0.04% at an RH of 95% for the new TD free base crystal, which means a hygroscopicity increase below 0.05% was observed (Figure 6). Accordingly, the comparative evaluation revealed that the moisture content increased over 1% dependently to the RH increment for TDF Form-I, while a relatively insignificant hygroscopicity could be observed for the new TD free base crystal. Thus, the new TD free base crystal is proved to be excellent for its high and prolonged storage and stability.

### 2.4. Stability Evaluation of TD Free Base Crystal and TDF Form-I under Accelerated and Stress Storage Conditions

**Figure 7:**
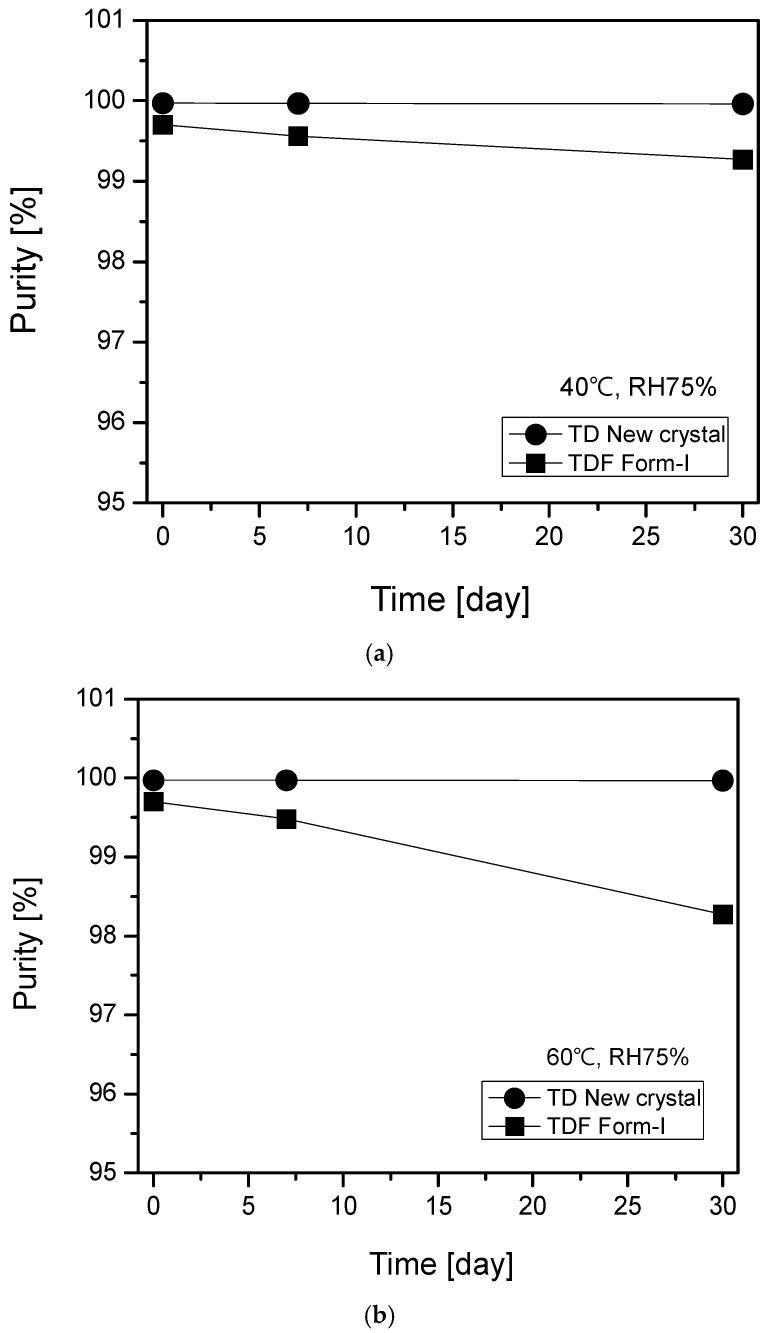

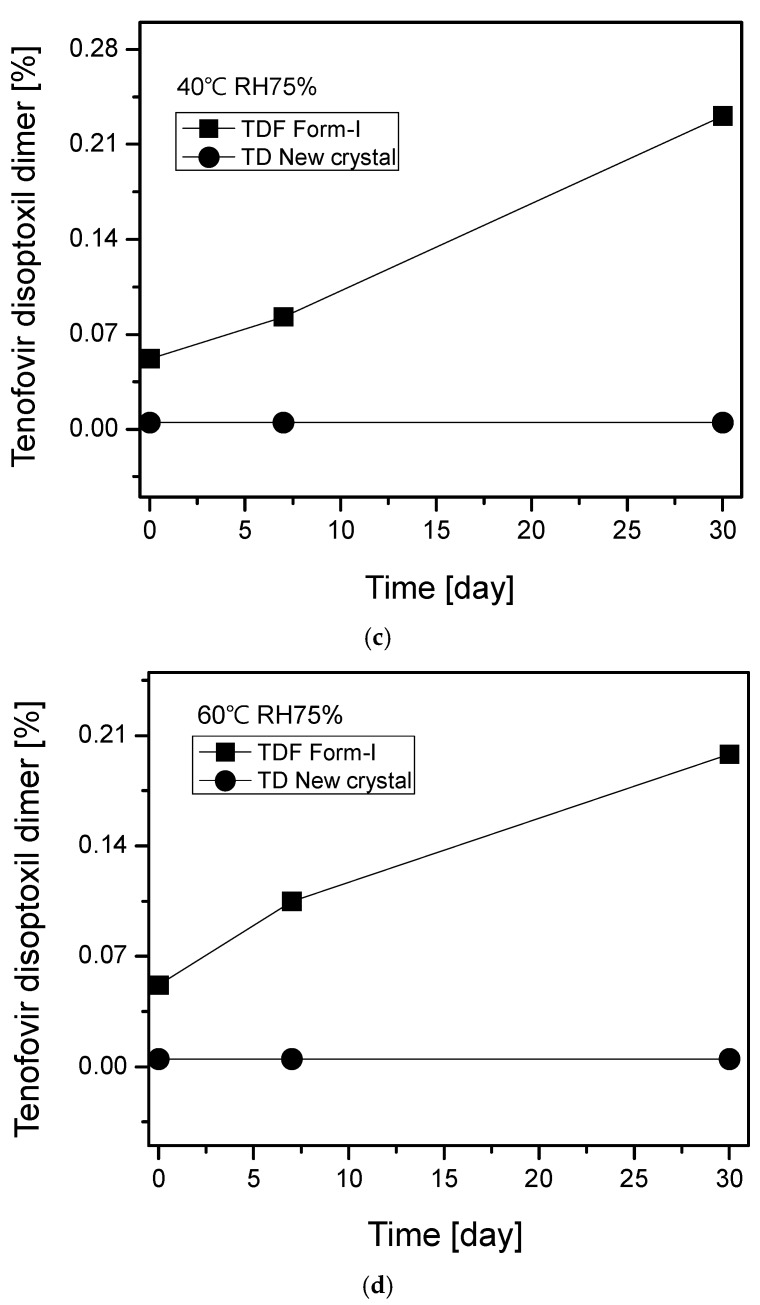
Comparative evaluation of the storage stability of and TD dimer formation from TD free base crystal and TDF Form-I under accelerated and stress conditions. (**a**) Storage stability at accelerated condition (40 °C, RH 75%), (**b**) storage stability at stress condition (60 °C, RH 75%), (**c**) TD dimer formation at accelerated condition (40 °C, RH 75%), (**d**) TD dimer formation at stress condition (60 °C, RH 75%).

In order to test the storage stability, TD free base and TDF Form-I were stored at a constant temperature and relative humidity for 30 days under accelerated (40 °C, RH 75%) and stress (40 °C, RH 75%) conditions, and thereafter were analyzed to determine and to compare the change in their purity after a prolonged storage period. By means of HPLC, the purity of the test samples was analyzed in triplicate, and then the average measurement was calculated in order to evaluate the storage stability of the samples. Under accelerated conditions (40 °C, RH 75%), the average purity value of TDF Form-I nudged down from 99.703% in the early stage, to 99.563% after 7 days, to 99.268% after 30 days. In contrast with TDF Form-I, no purity alteration was observed as the average purity values varied within the error range from 99.973% in the early stage, to 99.969% after 7days, to 99.961% after 30 days for the new TD free base crystal (Figure 7a). Furthermore, under stress storage conditions (60 °C, RH 75%), the average purity declined from 99.703% in the early stage, to 99.449% after 7 days, to 98.273% after 30 days for TDF Form-I on one hand, while, on the other hand, alike with the accelerated conditions, the average purity values of the new TD free base crystal varied within the error range from 99.973% in the early stage, to 99.97% after 7 days, to 99.968% after 30 days (Figure 7b). An additional analysis was conducted to investigate the probable formation of TD dimer from the new TD free base and TDF Form-I under the accelerated and stress storage conditions. The results revealed an increase of TD dimer from 0.052% at the beginning stage to 0.083% and 0.231% after 7 and 30 days, respectively, for TDF Form-I under the accelerated storage conditions. However, the same result showed that no impurity increase could be observed for the new TD free base, because the impurity value of 0.005% observed at the beginning was not changed either after 7 or 30 days (Figure 7c). Besides, under stress storage conditions, the TD dimer augmented from 0.052% at base to 0.105% and 0.198% after 7 and 30 days, respectively, for TDF Form-I. While, similar to the accelerated storage condition, no impurity increment could be observed for the new TD free base after 30 days (Figure 7d).

Accordingly, the new TD free base crystal is in more excellent form than TDF Form-I in terms of storage stability with high purity, as it produces absolutely minor impurity that is more optimal for long term storage.

### 2.5. Effect of Gastrointestinal pH on the Solubility of TD Free Base Crystal and TDF Form-I

Table 4 illustrates the solubility result of the TD free base crystal and TDF Form-I monitored under gastrointestinal pH variation. The experimental method is detailed in Section 3.8 of Materials and Methods. As presented in Table 4, the solubility of the TD free base crystal and TDF are similar in both distilled water (DW) and the buffer solutions. Accordingly, we could assess that the ionization effect has no influence on the solubility of TD free base crystal and TDF Form-I. From this study, rather than the currently available TDF Form-I, TD free base crystal is suggested as a potential API, owing to its enhanced hygroscopic stability.

## 3. Materials and Methods

### 3.1. Materials

Synthesized Tenofovir disoproxil (TD) and Tenofovir disoproxil fumarate (TDF) Form-I powders were provided by the Pharmaceutical Raw Material company J2H Biotech. Co., Ltd. (Ansan, Korea). Isopropyl alcohol (IPA) and *n*-hexane (Hex) were purchased from DaeJung Chem. Co. Ltd. (Seoul, Korea).

### 3.2. Drowning-Out Crystallization Method

Five hundred milligrams (500 mg) of TD free base oil and 5 mL of IPA were placed into a 20 mL vial and were dissolved at 25 °C. Afterward, 5 mL of Hex was added to a TD/IPA solution, and the mixture was allowed to stand without any agitation for 3 days. As a result, 400 mg of 0.24 × 0.10 × 0.08 mm-sized crystals were yielded. In addition, 500 mg of TD free base liquid was treated according to the crystallization method mentioned above, and then stirred at 200 rpm for 12 h to yield 400 mg of TD free base powder. A comparative study was conducted on the TD free base and TDF Form-I powder for the hygroscopic and stability evaluation. The powder and single crystals of the TD free base were used for a PXRD and DSC analysis, and the results revealed that both the powder and the crystalline forms are of an identical structure.

### 3.3. Differential Scanning Calorimetry (DSC)

DSC experiments were carried out on a DSC Q20 (TA Instruments, Philadelphia, PA, USA) using a Tzero pan under a purified nitrogen flow of temperature ranging from 20 °C to 150 °C and a scan rate of 10 °C/min.

### 3.4. Powder X-ray Diffraction (PXRD)

The PXRD analysis was performed on a Powder X-ray diffractometer (Bruker, D8 Advance, Billerica, MA, USA) with Cu Ka radiation at 45 kV and 40 mA and a scan rate of 3 °/min over a 2θ range from 5° to 35°. The divergence and scattering slits were 1°, while the receiving slit was 0.2 mm.

### 3.5. Single Crystal X-ray Diffraction (SXD)

The crystal structure of the TD free base was determined by the single crystal X-ray diffraction method. A preliminary examination and data collection were performed with Mo-*K*α radiation (λ = 0.71073 Å) on a Rigaku R-AXIS RAPID diffractometer (Rigaku Corporation, Tokyo, Japan). The unit cell parameters and the orientation matrix for the data’s collection were obtained from least-squares refinement, using the setting angle of 4478 reflections in the range of 6.0° < 2θ < 50.0° [18]. The crystallographic details are described in Table 2. Further intensity data were collected at 290(1) K with the *ω* scan technique. The intensity statistics and systematic absences are consistent with the orthorhombic space group *P*2_1_2_1_2_1_. The initial positions for all of the non-hydrogen atoms were obtained by the direct methods of the SHELXS-2013/1 program (2013/1, University of Göttingen, Germany) and Fourier methods [19]. The positions of the hydrogen atoms were idealized with the use of the riding model. The structure was refined by full-matrix least-squares techniques with the use of the SHELXL-2014/7 program (2014/7, University of Göttingen, Göttingen, Germany) in the WinGX program package. Refinements with the inverted structure gave flack x parameter 1.2(2), and the higher wR2 values and the current absolute structure were accepted. A different Fourier synthesis calculated with phases based on the final parameters shows no peak heights greater than 0.376 e/Å^3^. No unusual trends were found in the goodness-of-fit as a function of $F_{o}$, sinθ/λ, and the Miller indices. The final values of the atomic positional parameters, the equivalent isotropic displacement parameters, the anisotropic displacement parameters (ADP), and complete tabulations on the X-ray studies can be found in CIF format in the Supporting Information.

### 3.6. Hygroscopicity Evaluation Using Hygroscopic Tester

One hundred milligrams (100 mg) of the new TD free base powder and 100 mg of TDF Form-I were placed in a moisture test glass tube, and then were dried using nitrogen gas at 25 °C for 12 h. Afterward, by means of a hygroscopic tester (Quantachrome, Hydrosorb 1000, Boynton Beach, FL, USA), the rate of weight loss of the samples was automatically measured at a relative humidity (RH) of 15%, 35%, 55%, 75%, and 95% for 12 h.

### 3.7. Accelerated Stability and Stress Testing Analysis Using High-Performance Liquid Chromatography (HPLC)

Two hundred milligrams (200 mg) of each of the new TD free base powder (purity: 99.973%) and TDF Form-I (purity: 99.703%) samples were introduced into the stability chamber, and were stored for 30 days under accelerated (40 °C, RH 75%) and stress (60 °C, RH 75%) conditions in order to achieve a comparative storage stability. Afterward, the purity was determined by HPLC (Agilent 1100, Santa Clara, CA, USA). The analysis was performed using a C18 (4.6 × 150 mm, 5 μm, Kromasil^®^, Bohus, Sweden) column. The HPLC condition was H_2_O:MeOH:AN=10:7:3 (*v*/*v*/*v*) mobile phase and a flow rate of 1 mL/min, while the column temperature was set at 25 °C, and the detecting wavelength at 254 nm. The purity test evaluation was performed three times at the early stage, three times after 7 days, three times after 30 days, and then the average purity was calculated.

### 3.8. Method for the Solubility Test at Distinct Gastrointestinal pH

In order to perform a comparative evaluation of the TD free base crystal’s and TDF Form-I’s solubility at different gastrointestinal pH, buffer solutions of pH 1.2, 4.0, and 6.8 were prepared according to the Dissolution part for the preparation method of buffer solution in the USP regulations (Dissolution part for the preparation method of buffer solution). The buffer solution of pH 1.2 was prepared by dissolving 2 mg of sodium chloride and 7 mL of hydrochloric acid in 1 L of distilled water (DW). A pH 4 buffer solution was prepared by dissolving 3 g of acetic acid into 100 mL DW, adding 3.4 g sodium acetate to the prepared acetic acid solution, then supplementing the solution with 500 mL of DW. The buffer solution of pH 6.8 was achieved by dissolving 3.4 g of Monobasic Potassium Phosphate and 3.35 g of Anhydrous Dibasic Sodium Phosphate in 1 L of DW. All of the prepared solutions were adjusted using a pH meter. One hundred milligrams (100 mg) of TD free base crystal and TD Form-I were individually dissolved in the prepared buffer solutions, and were then agitated at 150 rpm for 3 days at ambient temperature. Afterwards, the solutions were allowed to settle for 3 h. The supernatant of each solution was collected for subsequent HPLC analysis. The HPLC analysis method was performed with reference to Section 3.7 of Materials and Methods.

## 4. Conclusions

In the present study, a TD free base single crystal was prepared with the use of drowning-out crystallization techniques. The purpose was to report the crystal structure of the TD free base as well as its solubility, which aspects of the TD free base remained currently unknown. On the other hand, the study aimed to overcome the storage stability drawback of TDF and afterwards to evaluate the potentiality of the TD free base as an API. The yielded TD free base crystal was characterized by means of PXRD, DSC, and video microscopes. Moreover, the crystal structure of the TD free base crystal was determined by single crystal X-ray diffraction, and the analysis’ results revealed that the crystal possesses a new crystal structure which has not been previously reported. Furthermore, a comparative storage stability evaluation was performed at accelerated (40 °C, RH 75%) and stress storage (60 °C, RH 75%) conditions for 30 days. The analysis’ result demonstrated the low impurity of the TD free base crystal compared with TDF Form-I. According to the analysis, the new TD free base has a crystalline form ideal for long term storage while preserving a high purity level. In addition, the comparative hygroscopicity evaluation for TDF Form-I and the new TD free base crystal shows that the moisture content increment for TD free base crystal was negligible. While TDF Form-I contains an acid which can make ionic pairs increase solubility in water, TD free base is a neutral molecule. We believe that these are responsible for the less hygroscopic nature of the TD free base compared with TDF Form-I. In addition, the intermolecular hydrogen bonds between N(1)-H---N(3) and N(1)-H---N(9) of the TD free base crystal are assumed to influence the decrease of the crystal’s polarity. The subsequent decrement of polarity prevents the crystals from hydrolyzing, which has as a direct consequence the improvement of the crystal’s hygroscopic property.

In addition, the experiment demonstrated that the TD free base has concurrent solubility with TDF Form-I under a gastrointestinal pH condition. In conclusion, the crystalline form of the TD free base is assumed to be a potential candidate to replace the hygroscopic TDF Form-I.

## Figures and Tables

**Table 1:** Crystallographic data for the known TDF crystals.

	Form-A [11,12]	Form-B [11]	Form-I [10]	ULT-11 [13]
TDF crystals	TD/FA 2:1 Co-crystal	TD/FA 1:1 Co-crystal MeOH solvate	TD/FA 1:1 Salt form	TD/FA 1:1 Salt form
Formula weight (g/mol)	1153.963	651.543	635.52	635.52
Crystal system	monoclinic	orthorhombic	monoclinic	monoclinic
Space group	*P*2_1_	*P*2_1_2_1_2	*P*2_1_	*P*2_1_
*a* (Å)	9.7774 (2)	18.438 (10)	9.835 (2)	9.7440 (3)
*b* (Å)	22.2104 (5)	34.057 (2)	22.315 (5)	18.0330 (5)
*c* (Å)	12.5000 (3)	9.907 (6)	12.545 (3)	17.4080 (6)
β (°)	95.1689 (1)	90	95.042 (3)	102.759 (2)
Cell volume (Å^3^)	2703.46	6221.03	2744.88	2983.3

**Table 2:** Crystal data and structure refinement for the Tenofovir disoproxil (TD) free base.

Parameter	TD Free Base Crystal
Chemical formula	C_19_ H_30_ N_5_ O_10_ P
Formula weight, amu	519.45
Crystal system	Orthorhombic
Space group	*P*2_1_2_1_2_1_
*a*, Å	8.7039 (5)
*b*, Å	12.2787 (8)
*c*, Å	23.8682 (16)
Cell volume (Å^3^)	2550.9 (3)
Z	4
Temperature, K	290 (1)
Radiation	Graphite monochromated MoKα (λ = 0.71073 Å)
Linear absorption coefficient, mm^−1^	0.168
Crystal size, mm^3^	0.24 × 0.10 × 0.08
Scan type	ω
θ limits, °	3.0° < θ < 25.00°
No. unique data	4478
No. unique data with I >2σ(I)	2541
wR2 (all data)	0.1706
R (on $F_{o}$ for I >2σ(I))	0.0667
Goodness-of-fit on *F*^2^	1.015
Flack x parameter	−0.13 (11)
Minimum and Maximum residual electron density (e/Å^3^)	−0.317 and 0.376

**Table 3:** Inter- and intra-molecular hydrogen bonds in Tenofovir disoproxil (TD) free base.

Type	Donor–H…Acceptor	D–H (Å)	H…A (Å)	D…A (Å)	D–H…A (°)
Inter	N(1)–H(1A)…N(9) **^i^**	0.86	2.22	3.083(8)	177
	N(1)–H(1B)…N(3) **^ii^**	0.86	2.26	3.099(8)	166
	C(8)–H(8)…O(17) **^iii^**	0.93	2.38	3.152(9)	141
	C(33)–H(33)…N(9) **^iv^**	0.98	2.58	3.510(11)	159
Intra	C(15)–H(15B)…N(5)	0.97	2.60	2.293(9)	129
	C(19)–H(19A)…O(17)	0.97	2.55	2.990(10)	108
	C(24)–H(24)…O(22)	0.98	2.39	2.745(14)	100
	C(28)–H(28A)…O(17)	0.97	2.54	3.006(10)	110

Symmetry codes: (i) −1/2 + x, 1/2 − y, −z; (ii) 1/2 + x, 1/2 − y, −z; (iii) 2 − x, −1/2 + y, 1/2 − z; (iv) −1 + x, y, z.

**Table 4:** pH dependent TD free base and TDF solubility.

Solubility (mg/mL)	TD Free Base	TDF
DW	5.88 mg/mL	6.2 mg/mL
pH 1.2	34.88 mg/mL	35.0 mg/mL
pH 4.0	5.12 mg/mL	5.12 mg/mL
pH 6.8	6.91 mg/mL	6.91 mg/mL

## Supplementary Materials

Supplementary materials are available online. CCDC 1505284 contains the supplementary crystallographic data for this paper. These data can be obtained free of charge via http://www.ccdc.cam.ac.uk/perl/catreq.cgi (or from the CCDC, 12 Union Road, Cambridge CB2 1EZ, UK; Fax: +44 1223 336033; E-mail: deposit@ccdc.cam.ac.uk)
